# Remodeling and Fibrosis of the Cardiac Muscle in the Course of Obesity—Pathogenesis and Involvement of the Extracellular Matrix

**DOI:** 10.3390/ijms23084195

**Published:** 2022-04-11

**Authors:** Jagoda Kruszewska, Agnieszka Cudnoch-Jedrzejewska, Katarzyna Czarzasta

**Affiliations:** Chair and Department of Experimental and Clinical Physiology, Laboratory of Centre for Preclinical Research, Medical University of Warsaw, 02-091 Warsaw, Poland; jg.kruszewska@gmail.com (J.K.); agnieszka.cudnoch@wum.edu.pl (A.C.-J.)

**Keywords:** cardiac fibrosis, cardiac remodeling, collagen, extracellular matrix (ECM), metalloproteinase (MMPs), obesity, tissue inhibitor of metalloproteinases (TIMPs)

## Abstract

Obesity is a growing epidemiological problem, as two-thirds of the adult population are carrying excess weight. It is a risk factor for the development of cardiovascular diseases (hypertension, ischemic heart disease, myocardial infarct, and atrial fibrillation). It has also been shown that chronic obesity in people may be a cause for the development of heart failure with preserved ejection fraction (HFpEF), whose components include cellular hypertrophy, left ventricular diastolic dysfunction, and increased extracellular collagen deposition. Several animal models with induced obesity, via the administration of a high-fat diet, also developed increased heart fibrosis as a result of extracellular collagen accumulation. Excessive collagen deposition in the extracellular matrix (ECM) in the course of obesity may increase the stiffness of the myocardium and thereby deteriorate the heart diastolic function and facilitate the occurrence of HFpEF. In this review, we include a rationale for that process, including a discussion about possible putative factors (such as increased renin–angiotensin–aldosterone activity, sympathetic overdrive, hemodynamic alterations, hypoadiponectinemia, hyperleptinemia, and concomitant heart diseases). To address the topic clearly, we include a description of the fundamentals of ECM turnover, as well as a summary of studies assessing collagen deposition in obese individuals.

## 1. Introduction

Obesity today has reached pandemic proportions, as excess weight may even be affecting up to two-thirds of the adult population in developed countries and is one of the main causes of disability worldwide [1,2]. It is a well-established risk factor for the development of metabolic disorders such as insulin resistance, diabetes, dyslipidemia, as well as cardiovascular diseases such as atherosclerosis, ischemic heart disease, myocardial infarction, hypertension, and atrial fibrillation [3,4,5,6,7]. It has been postulated that it may contribute to the development of heart failure with preserved ejection fraction (HFpEF) [8], as well as heart failure with reduced ejection fraction—HFrEF (usually by increasing the risk of myocardial infarction) [8]. Obesity cardiomyopathy is a term describing heart failure in the course of severe obesity of long duration, in which left ventricular hypertrophy (LVH) is the most common finding [8]. Importantly, obesity is associated with several hemodynamic changes and metabolic, inflammatory, and neurohormonal alterations such as increased activity of both the renin–angiotensin–aldosterone system (RAAs) and the sympathetic nervous system (SNS), hyperleptinemia, and hypoadiponectinemia, which may all have an impact on heart remodeling and heart function [2,9].

Cardiac remodeling is defined as a change in the size, shape, or structure of one or more of the cardiac chambers [9]. It results from changes in both the phenotype of the cardiomyocytes and the extracellular matrix (ECM) [10]. Excessive synthesis of ECM components, especially collagen, by residential fibroblasts in the myocardium, is known as fibrosis [11]. This process is inextricably associated with constant ECM turnover and may be related to an imbalance between ECM degradation by enzymes known as metalloproteinases (MMPs) and their specific inhibitors (tissue inhibitors of metalloproteinases; TIMPs) [12]. It also may lead to disruption of the cardiac architecture, enhancing its stiffness and, therefore, deteriorating the systolic or diastolic function and facilitating the occurrence of arrhythmia [11].

This review aims to provide a rationale for cardiac remodeling and fibrosis in the course of obesity, by highlighting the putative reasons for this process and summarizing studies focusing on assessing collagen accumulation in the ECM. We also comment on the association of obesity with several cardiovascular disorders, which may additively impact the cardiac geometry or are the consequence of adverse remodeling. To address this topic clearly, a concise description of ECM turnover is provided.

## 2. Materials and Methods

For the purpose of this review, we searched PubMed using the phrases ‘obesity and cardiac remodeling’, ‘obesity and cardiac fibrosis’, ‘obesity and cardiac ECM’, ‘high fat diet and cardiac remodeling‘, ‘high fat diet and cardiac fibrosis’, ‘high fat diet and cardiac ECM’, ‘leptin, ‘adiponectin’, ‘cardiac ECM’, ‘collagen’, ‘obesity and atrial fibrillation’, ‘obesity and heart failure’, and ‘obesity and hypertension’. We included review papers, original studies, and meta-analyses. The search was conducted up to March 2022.

Due to the extensiveness of the topic, we excluded studies that focused merely on the impact of type 2 diabetes mellitus on the cardiac muscle, as well as those assessing the influence of weight reduction on the myocardium.

## 3. Obesity as a Heterogenous Disorder

The definition of obesity is based on the body mass index (BMI) (calculated as the ratio of body mass in kilograms (kg) divided by the height in meters squared (m^2^)) and is diagnosed when the BMI value exceeds 30 kg/m^2^, whereas a person is described as overweight when their BMI value is 25–29.9 kg/m^2^ [13,14]. In children, obesity is diagnosed when the BMI value is above the 95th percentile adjusting for both age and gender [15,16]. According to WHO recommendations, the severity of obesity may be assessed byits division into classes, categorized by BMI: I class,30–34.9 kg/m^2^; II class, 35–39.9 kg/m^2^; and III class, over 40 kg/m^2^ [17]. The third class is also referred to as severe, extreme, or massive obesity [17]. The etiology of obesity is multifactorial, involving a complex interaction between genetical, hormonal, environmental, dietary, and behavioral factors; obesity develops when the caloric intake is disproportionately higher than the energy expenditure [18,19].

It must be acknowledged that obesity is a heterogenous disorder and there are a few possible phenotypes of obese subjects: metabolically healthy obese (MHO); metabolically abnormal obese (MAO); metabolically obese, normal weight (MONW), who are individuals characterized by a BMI < 25 kg/m^2^, but affected with complications such as hyperinsulinemia/insulin resistance, abdominal and visceral adiposity, unfavorable adipokine and lipid profile, and hypertension; and sarcopenic obese (SO) (whose body composition is made up of a high fat content and low muscle mass with an accompanying “normal” BMI) [20,21].

Obesity is reaching pandemic proportions and may trigger the development of serious metabolic and cardiovascular complications [22]. In fact, it is believed that the majority of the adverse effects of obesity on the cardiovascular system and mortality risk are not attributable to obesity itself but to the concomitant metabolic syndrome [3]. BMI is not always considered to be a good predictor of future health complications, as it is not a reliable measure to assess individual body fatness and adipose tissue distribution (visceral vs. subcutaneous adipose tissue) [23]. It must be remembered that excessive adipose tissue itself is also responsible for several hormonal and proinflammatory disturbances [24]. Due to several pathomechanisms, obesity may switch macrophage polarization towards the M1 phenotype, which is considered to be proinflammatory and profibrotic [8]. Furthermore, adipose tissue excess in obese individuals is associated with chronic subclinical inflammation and increased infiltrations of the immune cells [25]. Adipose tissue is also considered to be an active endocrine organ, as it secretes several hormones known as adipokines [26]. Recently, there has beena plethora of new emerging substances. In this review, we include two of the most common hormones, leptin and adiponectin, whose defective signaling in obesity can contribute to myocardial fibrosis.

Several experimental models were developed to study obesity-related complications. The most typically encountered models for obesity induction are animals with genetic alterations, for example, null for the leptin gene (ob/ob), with a mutation in the leptin receptor gene (db/db, fa/fa), or fed with laboratory chow with a high percentage of fat content (high-fat diet—HFD) [27]. In animal models, similarly to obese people, obesity is accompanied by several metabolic disturbances (for example, hyperlipidemia, hyperinsulinemia, and glucose intolerance) [27].

## 4. Distinctive Characteristics of the Cardiovascular System in the Course of Obesity

Obesity may affect the cardiovascular system via hemodynamic (increased workload) and non-hemodynamic factors (increased activation of the sympathetic nervous system (SNS) and the renin–angiotensin–aldosterone system (RAAs), insulin resistance/hyperinsulinemia, leptin insensitivity/hyperleptinemia, reduced adiponectin concentration/hypoadiponectinemia, overexpression of the peroxisome-proliferator-activated receptor (PPAR), decreased natriuretic peptides, lipotoxicity, oxidative stress, and chronic inflammation/hypoxia) [5,28,29].

The heart of obese individuals are characterized by cardiomyocyte hypertrophy, infiltration of fat into the ECM (steatosis), and accumulation of triglycerides among the contractile elements [28,30]. All of those factors impact the left ventricle (LV) geometry [28,30]. Moreover, the myocardium in obese people is surrounded by excessive epicardial adipose tissue (EAT) [8]. It has been shown that EAT may secrete several cytokines such as tumor necrosis factor α (TNF-α), interleukin 6 (IL-6), activin A, connective tissue growth factor (cTGF), MMPs, and angiopoietin-like 2 (ANGPTL2) [26,31,32]. Moreover, there aredata showing that increased EAT in the course of obesity may facilitate cardiac fibrosis and promote the incidence of arrythmia [32,33,34,35].

Another adverse impact of obesity on the myocardium is associated with lipotoxicity [36]. Physiologically, in the healthy myocardium, free fatty acids (FFA) constitute an elementary energetic substrate (approximately 70%) for the synthesis of adenosine triphosphate (ATP) [37]. In obesity, there is high availability of those substrates in circulatory form, which can directly interact with the PPAR receptors. Activation of PPAR receptors leads to increased uptake of FFA from the blood to the cardiomyocyte due to the CD36/fatty-acid transport protein (FAT) transportation to the cell membrane and stimulation of expression of enzymes necessary for FFA removal by β-oxidation in the mitochondria [38]. It was also shown that this process may be facilitated by leptin throughthe short-term observation [39]; such a prolonged condition may induce myocardial lipid accumulation [40].

The presence of triglycerides may not appear to be toxic;nevertheless, a lipid overload that exceeds the oxidative or storage capacity of the tissue may provoke the induction of other alternative biochemical pathways, for example, towards the production of ceramides, which may preserve proapoptotic properties [36,41,42]. Moreover, FFAs promote diacylglycerol (DAG) synthesis, which may activate protein kinase C (PKC), which in turn inhibits insulin signaling via the phosphatidylinositol 3-kinase/protein kinase B (PI3K/Akt)-dependent pathway [36,38]. Lipotoxicity also contributes to the excessive production of reactive oxygen species (ROS) and impaired mitochondrial biogenesis, which favor both inflammation and cellular apoptosis [43].

In the next section, we describe more comprehensively the hemodynamic factors, which are the main causative factor for cardiomyocyte hypertrophy in obese individuals [28]. Non-hemodynamic factors and their contribution to the changes in the cellular and ECM components will be presented in the paragraph entitled ‘The effect of obesity on myocardial ECM expression, heart fibrosis, and cellular hypertrophy’ in Section 7.

### 4.1. Hemodynamic Changes Observed in Obesity

**Figure 1:**
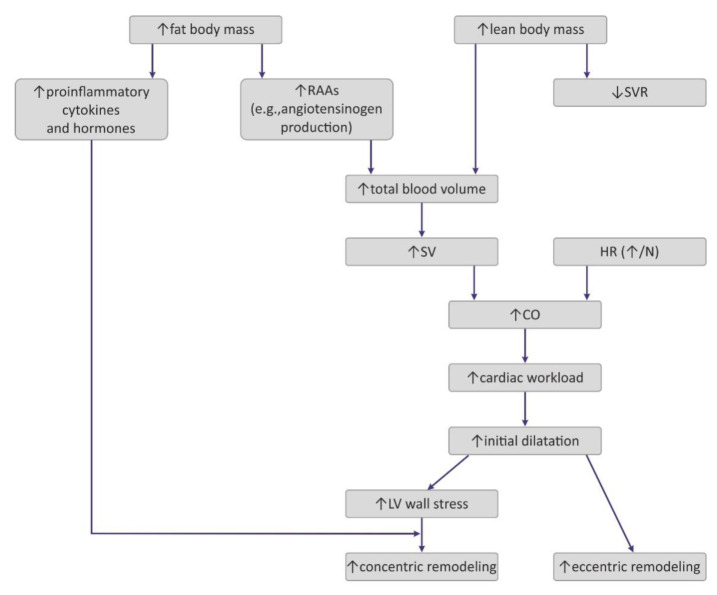
Pathogenesis of hemodynamic alterations in the course of obesity. RAAs—renin–angiotensin–aldosterone system; SVR—systemic vascular resistance; SV—stroke volume; HR—heart rate; CO—cardiac output; LV—left ventricle.

Obesity exerts several adaptive hemodynamic changes on the cardiac muscle (Figure 1) [44]. First, obesity is generally considered to be a hypercirculatory condition [45]. Elevated fat-free (lean) mass and the high activity of RAAs may be putative reasons [9,44,45]. In obese patients, increased cardiac output (CO) is observed, and as the heart rate (HR) remains normal or may be only slightly elevated, a major cause for this high CO is subjected to increased stroke volume (SV) [9]. Increased SV may also result in increased LV end-diastolic pressure, which was also detected in obese persons [9,44].

Interestingly, excess weight leads to decreased systemic vascular resistance (SVR), which may also lead to the augmentation of CO and LV dilatation [9]. Increased chamber volume exerts tension on the ventricular wall, according to the Law of Laplace, and such a chronic process may cause hypertrophy [29]. Such remodeling depicts the model of volume overload that contributes to eccentric hypertrophy, characterized by the lengthening of the myocytes [46].

Clinical manifestation of hemodynamic changes in the course of obesity may differ entirely depending on the distribution of the adipose tissue in the body [47]. The Dallas Heart Study carried out on 2710 participants without organic heart disease suggested that hemodynamic features such as high CO and low SVR may pertain to individuals with an excess of subcutaneous adipose tissue (SAT), especially distributed within the gluteal-femoral region, whereas increased visceral adipose tissue (VAT) was rather related to lower CO and elevated SVR [47]. Patients with excessive VAT proportions developed a concentric hypertrophy of LV (i.e., increased myocyte thickness), and also had increased LV wall thickness, increased LV mass/volume ratio, and smaller LV end-diastolic volume [46,47]. Similarly, Liu et al. showed in their study that VAT in obese patients was a better predictor of cardiac remodeling and subclinical dysfunction of LV than elevated BMI [48].

### 4.2. The Impact of Obesity on Myocardial Geometry and the Ejection Fraction

Left ventricular hypertrophy appears to be the most predominant observation in the hearts of obese individuals [44,49,50,51]. The results from the Framingham Heart Study, which was carried out on 3922 healthy participants, concluded that BMIwas strongly correlated with left ventricular mass, LV wall thickness, and LV internal dimensions [50]. In a different study, an increase in BMI by 1 kg/m^2^ and an increase in waist circumference by 1 cm increased the risk of LV hypertrophy by 5.1% and 2.6%, respectively [52]. It may be concluded that increased LV mass is commonly encountered among obese individuals and, in particular, it may be dependent on the central distribution of fat depots (abdominal fat) [53].

Nevertheless, in the literature, it is commonly discussed whether heart enlargement in the course of obesity results from concentric or rather eccentric hypertrophy [54]. A meta-analysis that included 4999 obese individuals and 6623 nonobese controls showed that eccentric hypertrophy was more frequent in obese subjects than the concentric phenotype [29,55]. This is in agreement with the initially formulated hypothesis of cardiomyopathy of obesity, based on increased cardiac output and blood volume, which in turn exerts tension on the LV wall, leading to its dilatation [56].

**Figure 2:**
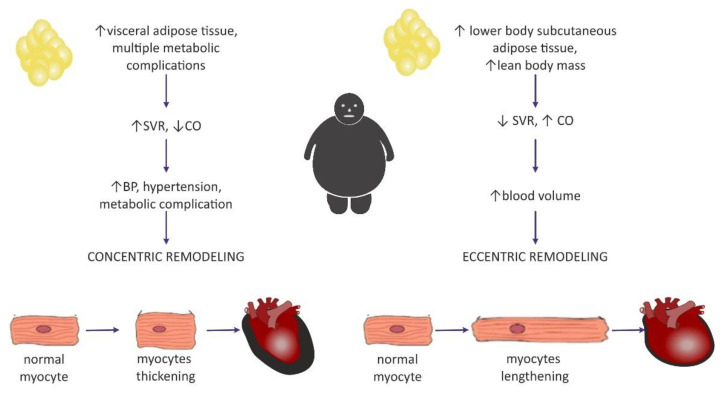
Two distinctive patterns of cardiac remodeling in the course of obesity and a proposition for their pathogenesis based on adipose tissue distribution. SVR—systemic vascular resistance; CO—cardiac output.

Today, investigators are more inclined to think that the concentric pattern may be more prevalent than initially expected [9,29] and is even independent of the occurrence of hypertension [54]. It is also believed that the distribution of fat tissue in the viscera was more correlated with concentric hypertrophy [29], as well as with higher blood pressure (BP) values in comparison with healthy, nonobese subjects, even without a diagnosis of hypertension, which may predispose to such remodeling [9]. Fat distribution and hemodynamic alterations also have an impact on cardiac remodeling, as mentioned above. Lower body SAT prompted eccentric cardiac remodeling, whereas abdominal subcutaneous adipose tissue did not have an effect on the hemodynamics. It was concluded that visceral abdominal adiposity (more often associated with the presence of metabolic syndrome) was a causative factor for concentric remodeling [47]. The existence of two different patterns of hemodynamic alterations in obese individuals may contribute to different LV remodeling (Figure 2).

While considering LV diastolic dysfunction, it was shown that it is present in all classes of isolated obesity [1,57,58]. Moreover, its degree correlated with BMI [57]. This supports the high prevalence of HFpEF in obese individuals [59].

Data about the systolic LV function are inconsistent, as various authors have reported that the LV ejection fraction (EF) was decreased, normal, or even supernormal in obese subjects [27]. An increased EF may be observed in the early stages of obesity due to increased volume overload [57].Some authors believe that long-lasting obesity without metabolic and cardiovascular comorbidities may not be conducive to the impairment of the systolic function [1,58]. Khan et al. concluded that there is not enough evidence in clinical studies to make the claim that obese patients have left ventricular ejection fraction (LVEF) < 35% [58]. Overt systolic dysfunction may suggest the presence of concomitant heart disease, especially coronary artery disease (CAD) [1,58].

Similarly, the right ventricle (RV) in obese individuals may by characterized by a mild increase in size and wall thickness [60]. In addition, a mild dysfunction of RV was reported [8]. This may be a potential reason for the high prevalence of sleep apnea among obese individuals [9]. Nevertheless, Wong et al. concluded that increased BMI was associated with the severity of RV dysfunction in overweight and obese subjects without overt heart disease, even independent of sleep apnea [60]. Moreover, obesity may also induce the enlargement of the left atrium (LAE) as a consequence of LV diastolic dysfunction [61,62]. The large MONICA/KORA study conducted on 1212 men and women showed that the presence of obesity was the strongest predictive factor for LAE development [63] Lavie et al. presumed that obesity may constitute a risk factor for atrial fibrillation [5].

To summarize, we may observe in obese individuals LV hypertrophy, either eccentric or concentric, LAEand dilated RV, as well as RV hypertrophy [29].

## 5. Myocardial Extracellular Matrix

The myocardial extracellular matrix (ECM) is a network of fibrillar collagens (mainly type I and III) and other nonfibrillar components such as glycoproteins—for example, fibronectin, proteoglycans, and glycosaminoglycans (GAG)—that surrounds the cardiac myocytes alignment and provides a connection between the cardiomyocytes, as well as between the cardiomyocytes and the surrounding vessels [64,65]. Such scaffolding converts the force generated by individual cardiomyocytes during the systole into an organized ventricular contraction and prevents cardiomyocyte slippage as well as overstretching during the diastole by providing passive stiffness [64,66]. It also influences the cardiac tissue architecture and chamber geometry [66]. Moreover, the interstitial network of connective tissues may also play a role in the mechanosensory process by intercellular signaling, such as through the collagen–integrin–cytoskeleton–myofibril connection [67]. Apart from being a cellular scaffolding, the ECM also constitutes an environment for numerous bioactive signaling molecules, such as transforming growth factor beta (TGF-β), TNF-α, angiotensin II (Ang II), and endothelin-1 (ET-1) among others [68]. They are often stored in inactive forms until they are activated in response to physiological or pathological stimuli [67].

Myocardial ECM undergoes constant turnover, approximately by 0.6% per day, physiologically [69]. Its composition is precisely regulated by MMPs and TIMPs, which are mostly synthesized by cardiac fibroblasts, as well as other cells such as cardiomyocytes, endothelial cells, and macrophages [70,71].

### 5.1. Collagen

Collagen is the main component of the cardiac ECM [72]. Recent morphometric evaluations of human hearts from the deceased for noncardiac reasons showed that collagen constitutes on average 15.2% of the RV, 8.6% of the interventricular septum (IVS), and 9.5% of the LV [73]. Generally, collagens, based on their structure, can be divided into two main classes: (1) fibril-forming collagens, which include the following types—I, II, III, and V; (2) nonfibrous collagens—type IV (which is the main component of the basal lamina) and type VI collagen [74]. In the cardiac ECM, fibrillar collagens types I and III are the most predominant, while collagen types IV (membrane base forming), V, and VI occur less abundantly [71]. Type I accounts for approximately 80% forms of all thick fibers and provides tensile strength in the myocardium, whereas type III collagen constitutes less than 10% of all collagens and forms a thin network of fibers that support distensibility of the heart [69,75]. The fibers are organized into the following areas: the epimysium, perimysium, and endomysium [66]. The epimysium is a sheath of connective tissue surrounding the entire muscle, whereas the perimysium surrounds groups of myocytes and the endomysium interconnects individual cells [76].

It has been shown that the amount of collagen fibers, their distribution, and organization are the determinators of heart function and alterations in its interface, both in structure and composition, may influence LV geometry and impair systolic and diastolic heart function [64,77].

Increased collagen accumulation in the ECM may appear as a sign of fibrosis within the heart muscle [71]. Beyond measurement of its protein levels, biomarkers of its synthesis and degradation are frequently assessed as indicators for collagen turnover [67,71]. Propeptides from the amino- and carboxy-terminal procollagen sides, which are cleaved in the ECM, are considered to be biomarkers of collagen synthesis. These are PICP (procollagen type I carboxy-terminal propeptide) and PINP (procollagen type I amino-terminal propeptide) for collagen type I, and their counterparts for collagen type III—PIIICP and PIIINP, and they are released in a stoichiometric manner [67,71]. During pathological ECM remodeling as well as physiological ECM turnover, collagen fibers are degraded, which is associated with the cleavage of C- and N-terminals of collagen molecules [67]. Hence, those C- and N-terminal telopeptides of collagen type I (CITP, NITP) and type III (CIIITP, NIIITP) are considered to be biomarkers of their degradation [67,71]. It is also feasible to assess the enzyme involved in collagen processing, such as prolyl-4-hydroxylase (PH4), procollagen-lysine,2-oxoglutarate 5-dioxygenase (PLOD), and lisyl oxidase (LOX) [78].

Not only does the amount of collagen have an influence on the activity of the heart muscle, but also the cross-linking of its fibers [79,80]. In most studies, the degree of cross-linking was determined by the amount of insoluble collagen versus soluble collagen in the heart [79]. The disturbances of cross-linking were observed in chronic diseases, which may be due to obesity-related comorbidities such as hypertension [81,82], chronic ventricular volume overload [83,84], diabetes [85,86] and in aging hearts [87]. Increased crosslinking may also contribute to enhanced diastolic stiffness of LV [87]. Furthermore, the reduction of cross-linking, regardless of its type and quantity may contribute to cardiac dilatation, which was observed in models of pressure-overload-induced heart failure [82].

Another factor worth considering is the ratio of type I collagen to type III collagen (I/III collagen ratio), as its increase may be responsible for left ventricle stiffness and a lower rate of relaxation [88,89]. Its elevation was observed in hypertension [79,89,90], in patients with dilated cardiomyopathy [91], obesity [92,93], and in the experimental model of myocardial infarct [94]. In diabetes, the contrary was observed, as this ratio was lower in diabetic animals and humans compared with unaffected controls [86,95].

### 5.2. Metalloproteinases (MMPs)

We distinguish two principal types of MMPs: MMPs that are soluble in the ECM and secreted in the latent proenzyme form (proMMPs) and membrane-type metalloproteinases (MT-MMPs, such as MMP-14, also known as MT-MMP-1) that undergo processing in the cellular compartment and, subsequently, are attached to the cell membrane in the already activated form [77,96,97].

Soluble MMPs encountered in the myocardium and involved in remodeling include interstitial collagenases such as MMP-1, MMP-8, MMP-13;the stromelysins such as MMP-3; and the gelatinases such as MMP-2, MMP-9, and MMP-28 also known as epilisyn [71]. MMP-1, MMP-8, and MMP-13 degrade type I, II, and III collagens. In addition, MMP-1 degrades the basement membrane proteins [71]. The classically known gelatins, MMP-2 and MMP-9, also process many collagens, including type I, IV, and V collagen; MMP-2 additionally cleaves type III collagen [12,98]. MT1-MMP can cleave many ECM proteins, including fibronectin, laminin-1, and type I collagen [71].

Expression and activity of MMPs is tightly controlled on many different levels [97,99]. First, it is regulated on a transcriptional level by a variety of growth factors, cytokines, chemokines, hormones, cellular transformation, and interaction with extracellular matrix components. Second, most MMPs (except the membrane type) are synthesized as inactive zymogens, called proMMPs [12], which require proteolytic activation by other already active MMPs or endogenic proteases such as plasmin, urokinase-type plasminogen activator (uPA), tissue plasminogen activator (tPA), or thrombin [64]. Third, active MMPs may be inhibited directly by their most specific inhibitors, such as tissue inhibitors of metalloproteinase (TIMPs) [12].

### 5.3. Tissue Inhibitors of Metalloproteinase (TIMPs)

TIMPs are low-molecular-weight proteins (21–30 kDA) that create noncovalent high-affinity complexes with active MMPs in the stoichiometric 1:1 ratio [12]. To date, there have been four TIMPs (TIMP-1, -2, -3, -4) reported in the literature [100]. All four of them are expressed in the normal human heart, but their profile varies under pathological conditions [101]. Beyond their apparent inhibitory properties towards MMPs, TIMPs are also involved in several other processes and may promote cellular growth, proliferation, and apoptosis [102]. For example, it has been shown that upregulated TIMP-1 may induce collagen synthesis and its elevated serum concentration may correlate with cardiac fibrosis [103].

## 6. Fundamentals of Heart Fibrosis

Fibrosis may be one of the components of cardiac remodeling and is a term referred to as the excessive accumulation of collagen within the ECM [11]. Fibrosis is a major risk factor of cardiac failure as well as a crucial determinant of myocardial function, diastolic stiffness, and a propensity for reentry arrhythmias. Increased fibrosis may reduce the elasticity and compliance of the ventricle, leading to diastolic dysfunction, and may induce arrhythmia and diminish oxygen availability by disrupting perfusion to the myocytes [70,71]. Such a chronic condition may be a predisposition to heart failure and may induce a different enzymatical response in the ECM [46,70,104].

Two types of fibrosis are usually distinguished: ‘reparative’ (that aims to replace necrotic heart tissue, for example, in ischemic injury or directly after myocardial infarction, MI) and ‘reactive’ (developing as a consequence of pathological hyperactivity of fibroblasts, encountered usually in nonischemic cardiomyopathies or occurring after MI in surviving heart muscle, or developing as a consequence of other injurious stimuli such as pressure overload, aging, or metabolic disturbances) [105]. Reactive fibrosis may further be divided into perivascular (expansion of the microvascular adventitia) and interstitial (expansion of the endomysium and perimysium space, accompanied by the accumulation of ECM proteins and significant cardiomyocyte loss) [70].

It has been shown that fibroblasts at the cellular level are responsible for the process of cardiac fibrosis. In the healthy heart, fibroblasts are rather quiescent and nondividing [26], whereas in myocardial fibrosis, they excessively proliferate leading to collagen turnover dysregulation, as evidenced by the domination of its synthesis over its degradation [106]. Such pathologic activation of fibroblasts may be due to growth factors such as TGF-β; platelet-derived growth factor (PDGF); epidermal growth factor (EGF); fibroblast growth factor (FGF); cytokines IL-1, IL-4, TNF-α;AngII;and aldosterone [71].

Myofibroblasts (myoFBs) are cells that do not exist in the healthy myocardium. Cardiac fibroblasts differentiate into myofibroblasts after injurious stimulus [107]. They possess a greater ability to produce a higher amount of ECM components and they have contractile properties preventing dilatation via cell–cell and cell–matrix interaction [71]. MyoFBs are generally considered to be a marker of the excessive deposition of ECM and express α-smooth muscle actin (α-SMA), which is a marker of pathologic fibroblast activity [108].

The best-characterized cytokine contributing to the process of fibrosis is TGF-β, which is secreted among others within macrophages and activates the SMAD signaling pathway in cardiac fibroblasts, which induces collagen synthesis [109]. TGF-β is secreted into the ECM as an inactive latent molecule (pro-TGF-β), which can be processed in the ECM to its active form by several proteases, including plasmin, MMP-2, MMP-9, and ROS [110,111]. TGF-β signaling occurs via assembling two dimeric TGF-β receptors (TGFβIR and TGFβIIR) [110]. Signal propagation by a cytoplasmic kinase domain of type I receptors (TGFβIR) leads to phosphorylation of Smad2 and Smad3 proteins, which are known as receptor-regulated Smad proteins (R-Smad) that act directly as transcription factors [110,112]. Translocation of Smad2/3 to the nucleus is facilitated by creating a complex with Smad4 (common Smad). Importantly, signaling via TGF-β type II receptor (TGFβIIR) may activate non-Smad signaling pathways, such as p38MAPK and JNK [110,111].

Importantly, total Smad 2/3 expression remains unchanged, as TGF-β rather promotes its activation via the phosphorylation of the downstream Smad 2/3 protein [113]. The role of TGF-β in ECM remodeling is quite well-established, as it can downregulate MMP9 expression and upregulate TIMP-1, prompt fibroblast differentiation into myoFBs, and increase the apoptosis of cardiomyocytes by directly stimulating mitogen-activated protein kinase (MAPK) p38 and c-Jun N-terminal kinase (JNK) in a Smad-independent manner [102,114].

It is not only the hyperactivity of fibroblasts that participates in the pathogenesis of cardiac fibrosis, but also the constant turnover of enzymes in the ECM. For example, proteolysis of the ECM by MMPs may promote fibroblast migration in response to cytokine release [71]. Interestingly, the activity of some MMPs, including MT1-MMP, MMP-2, and MMP-9, may also induce fibrosis through cleaving and activating latent ECM-bound TGF-β, which subsequently results in the activation of the SMAD pathway in cardiac fibroblasts and may trigger collagen production [115,116,117].

## 7. The Effect of Obesity on Myocardial ECM Expression, Heart Fibrosis, and Cardiac Hypertrophy

Experimental studies confirmed increased fibrosis and adverse remodeling in animal models of diet-induced obesity [92,118,119,120,121]. Czarzasta et al. observed increased fibrosis in Sprague Dawley rats exposed to the administration of a HFD for 12 and 16 weeks in comparison with controls, as well as a decrease in the cross-sectional area of LV cardiomyocytes in relation to the entire LV area, which can correspond to an increased apoptosis rate of cardiomyocytes due to lipotoxicity [118]. In another study, the same researchers confirmed that a HFD may alter the expression of JNK and p38 MAPK kinases [114]. In a study by Jimenez-Gonzalez et al., male Wistar rats fed with a HFD developed obesity, cardiac hypertrophy, and fibrosis, accompanied by triglyceride and ceramide accumulation in the cardiomyocytes [122]. Consistent results about enhanced collagen deposition were presented by other authors [92,121,123,124,125].

An interesting observation was reported by da Silva et al., who investigated increased collagen type 1 deposition in the cardiac ECM of Wistar rats after 15 weeks of HFD. However, after 30 weeks, collagen type 1 expression was profoundly diminished, even in comparison with the control group. Collagen type III expression was unaffected [126]. Decreased collagen type I was also detected in another study by the same researchers, in which HFD administration lasted 34 weeks [127]. Both studies proposed that prolonged obesity may impact ECM turnover differently, not in the matrix-preserving pattern [126,127].

Table 1 summarizes data on the impact of HFD administration in rodent models of ECM turnover. We only present selected data that have been proved to be statistically significant, where there was a comparison of animals fed on a HFD with animals fed on a normal diet as a control.

New Zealand White rabbits fed with a cholesterol-enriched diet also developed left ventricular diastolic dysfunction which was accompanied by changes in the ECM, such as increased mRNA for TGF-β and collagen type I, but not type III; hence, the I/III collagen ratio was increased. Hypercholesteremia in rabbits also resulted in increased mRNA for the vascular cell adhesion molecule-1 (VCAM1), MMP-12, TIMP-1, and IL-1 β [93].

Zhu et al. reported that a high-cholesterol diet (HCD) in pigs increased the collagen type I/III ratio, TGF beta expression, and the activity of its mediator Smad as well as cellular apoptosis through caspase-independent cascade [113]. After 12 weeks of HCD administration, the pigs exhibited diastolic dysfunction whereas no systolic impairment was observed [113].

Although there is increasing evidence that obesity may induce collagen deposition, little is still known about alterations occurring in the cardiac ECM in the course of obesity, especially in terms of metalloproteinase activity [70]. In the next paragraph, we include information about putative factors contributing to fibrosis and cardiac remodeling.

### 7.1. Neurohormonal Changes in Obesity Attributable to Heart Remodeling

#### 7.1.1. Sympathetic Nervous System (SNS)

Increased SNS activity is observed in obese individuals [5]. There are various hypotheses elucidating its overdrive including hypoxemia, hyperleptinemia, and insulin resistance [6].

Importantly, beta-adrenergic receptors are located on the cardiac fibroblasts and there are studies indicating that their stimulation may result in fibrosis and cardiac remodeling [130]. Jaffre et al. showed that beta-adrenergic receptor (β-AR) overstimulation of the cardiofibroblasts transactivated the protease activated receptor 1 (PAR1) through MMP-13, leading to the activation of the pathological Gαq pathway in cardiomyocytes, and both Gαq and ErbB in cardiac fibroblasts, predisposing cardiac dysfunction [131]. Similarly, chronic β_2_-adrenergic receptor (β_2_-AR) stimulation enhanced the proliferation of cultured human cardiac fibroblasts [132]. Moreover, transgenic mice with β_2_-AR overexpression exhibited greater interstitial fibrosis [133].

#### 7.1.2. Renin–Angiotensin–Aldosterone System (RAAs)

Enhanced RAAs activity is also observed in obese animals and people, which can be elucidated by the fact that local synthesis of angiotensinogen, angiotensin I, and angiotensin converting enzyme occurs in the adipocytes additively to their systemic renal production [9,134,135]. Moreover, adipokines (for example, leptin), as well as SNS activity may increase the RAAs activity. The adverse effect of the latter may involve the induction of hypertension, myocardial hypertrophy, and an increase in preload and afterload [9]. The RAAs components are also well-established factors leading to cardiac fibrosis [136,137,138,139]. Angiotensin II can bind to its type I receptor (AT1-R), which can activate cellular signaling pathways, including those involving extracellular signal-regulated kinase (ERK) and Janus family kinase (Jak). Subsequently, those pathways may induce the expression of c-fos, c-jun, and downstream proteins such as transforming growth factor-β1 (TGF-β1) and Smad3 [70,137]. Angiotensin II is also considered to be a growth factor, which contributes to cardiac hypertrophy in a TGF-β-activated kinase 1 (TAK1)-dependent manner [139]. Moreover, it may upregulate TIMP-1 expression [103].

#### 7.1.3. Natriuretic Peptides

Atrial and brain natriuretic peptides (ANP, BNP) usually correlate with the degree of heart failure and increase in response to increased atrial and ventricular stretching by volume overload [140]. Interestingly, in obese individuals, both peptides are found in comparatively lower concentrations [7]. It was shown that adipose tissue may also secrete neprilysin—an enzyme that degrades natriuretic peptides [141].

In terms of alteration in the ECM, ANP and BNP were shown to reduce vasoconstriction, inhibit fibroblast proliferation and differentiation into myofibroblasts, and inhibit collagen synthesis and MMP release via activation of the cyclic guanosine monophosphate (cGMP) pathway [142].

#### 7.1.4. Hyperinsulinemia and Insulin Resistance

Hyperinsulinemia and insulin resistance are inseparable complications of obesity [3]. Excessive adipose tissue secretes several factors such as proinflammatory cytokines, hormones, glycerol, and nonestrified fatty acids (NEFA) [143]. In the course of obesity, increased uptake of NEFA by peripheral cells leads to their intracellular accumulation and competition with glucose for substrate oxidation [143,144]. Consequently, enzymes required for glucose utilization (e.g., pyruvate dehydrogenase, phosphofructokinase, and hexokinase II) are inhibited [143,144]. Activity of several proinflammatory cytokines and hormones further aggravates insulin intercellular signaling, leading to the condition known as insulin resistance [145]. Pancreatic β-cells increase insulin secretion in order to overcome reduced sensitivity of peripheral tissue, leading to hyperinsulinemia [143].

It has been shown that it is also one of the factors putative for cardiomyocyte hypertrophy via overactivation of PI3K/AKT and RAS/RAF/MEK/ERK signaling [146]. In the study by Schiekofer et al., chronic Akt1 activation in transgenic mice was conducive to extensive cardiac hypertrophy, contractile dysfunction, and interstitial fibrosis [147]. Moreover, phosphorylation (i.e., activation) of AKT and its downstream mediators, glycogen synthase kinase-3 α and kinase-3 β (GSK3A and GSK3B), was further increased by additional β-adrenergic stimulation with ISO, which resulted in increased collagen accumulation [146]. It must be acknowledged that Akt signaling is considered to be a physiological pathway in reference to heart growth; nevertheless, there aredata suggesting that its prolonged activation in the course of obesity may exert a harmful effect on the myocardium [146,147].

### 7.2. Influence of Obesity-Related Tissue Inflammation and Hypoxia on Myocardial ECM Expression and Heart Fibrosis

Obesity is associated with hypertrophy of the adipocytes, which contributes to the development of a chronic hypoxic state [8].In healthy individuals, enlargement of the adipose tissue usually occurs via de novo adipogenesis from precursor cells, whereas adipose tissue expansion in the pathological obese also results from the enlargement of existing adipocytes [148]. This may trigger local hypoxia, in response to which adipose tissue macrophages may secrete hypoxia-inducible factor-1 α (HIF-1α) [8]. Hypoxia is also considered to be a missing link between obesity and systemic low-grade inflammation [149]. HIF-1α induces systemic inflammation, contributing to an increased concentration of IL-6, monocyte chemoattractant protein-1 (MCP-1), TNF-α, IL-1β, thrombospondin, pro α2 (I) collagen, TGF-β, nicotinamide adenine dinucleotide phosphate (NADPH) oxidase, and cTGF [108].

In the myocardial vasculature, proinflammatory cytokines upregulate adhesion molecules such as VCAM and E-selectins, which facilitates leukocyte (monocyte) subendothelial passage. Accumulation of inflammatory cells is conducive to alterations in coronary microvascular endothelial cells and ROSproduction [150]. Oxidative stress results from an imbalance between the antioxidant defenses and ROSproduction [151] and may be aggravated by impaired mitochondrial biogenesis in the cardiomyocytes of obese individuals. It activates nicotinamide adenine dinucleotide phosphate oxidase, causing nitrative stress, which limits nitric oxide (NO)bioavailability [150]. This, in turn, decreases the activity of the protein kinase G (PKG) in the adjacent cardiomyocytes causing their hypertrophy and the resultant hypophosphorylation of titin, which increases myocardial stiffness [150]. This mechanism is believed to serve an important role in the pathogenesis of HFpEF [150].

The available data suggest that the development of a chronic subclinical inflammatory condition in the course of obesity contributes to increased myocardial fibrosis. For example, differentiation of monocytes into macrophages and especially their polarization towards the M1 phenotype promotes the TGF-β-mediated fibrogenic program [8].

HIF-1α was also studied in terms of its contribution to increased fibrosis, and it has been shown that it may trigger the deposition of collagen type I, III, and IV and upregulate TIMPs and LOX [108]. Moreover, it also stimulates the expression of several cytokines and growth factors that target the proliferation and differentiation of fibroblasts such as FGF, PDGF, and connective tissue growth factors [152]. There are also studies that pointed to the involvement of HIF-1α in ECM regulation, as it may upregulate the expression of MMP-2 and MMP-9 [108,153].

### 7.3. The Effect of Selected Adipokines on Myocardial ECM Expression and Heart Fibrosis

Described below are leptin and adiponectin, two adipokines thatwere examined the most in terms of heart remodeling.

#### 7.3.1. Leptin

Leptin is an adipocyte-derived, 167-amino-acid hormone, initially known for its anorexigenic properties and impact on the hypothalamic area [154]. It is predominantly secreted by the adipose tissue (visceral, subcutaneous, and epicardial), but also by other organs, including the myocardium [39]. Today, it is known that its receptors (LepR a-f) are extensively distributed not only in the hypothalamus, but in various organs, including the heart [39].Cardiomyocytes express two isoforms of LepR (LepRa and LepRb) [155]. Leptin may exert its effect by direct binding to its receptors, or its activity may be mediated centrally by the SNS [156]. Leptin’s circulating levels correlate with adipose tissue mass [157]. Moreover, almost two times more leptin mRNA was detected in obese individuals than in lean counterparts [158]. Hyperleptinemia is a term assigned to describe the chronic increase in serum levels of leptin. It is believed that hyperleptinemia in obese subjects is not sufficient to overcome metabolic dysregulation, hence the hypothesis of leptin resistance [159].Whereas there is more evidence for hypothalamic leptin resistance, less is known about disturbances in its signaling in the peripheral tissue and myocardium [159].

Increased leptin concentration can impact the hearts of obese individuals by affecting oxidative stress, fatty acid uptake and oxidation, cardiomyocyte hypertrophy, and fibrosis [160]. Short-term incubation of HL-1 cardiomyocytes with leptin resulted in the enhanced oxidation of fatty acids and the transient reduction of lipid content [161]. However, prolonged exposure of the cardiomyocytes to leptin impeded FFA oxidation and induced FFA accumulation in the cardiomyocytes and lipotoxicity [161]. In terms of leptin-induced cardiac hypertrophy, both studies by Zeidan et al. on rat neonatal ventricular myocytes cultured for 24 h with 3.1 nmol/L of leptin showed that leptin may promote actin cytoskeleton reorganization leading to the translocation of p38 MAPK from the cytosol to the nuclei. This process was shown to be dependent on the signaling via the G protein Ras homolog gene family (Rho) and its downstream effector, the Rho-associated coiled-coil-forming protein kinase (ROCK) [162,163]. Thus, leptin-induced signaling via p38 MAPK may contribute to the increased cardiomyocyte area (hypertrophy) [162,163].

Leptin is also believed to contribute to ECM turnover and collagen synthesis [127,164,165,166]. Schram et al. conducted a study on neonatal rat cardiac myofibroblasts and showed that their incubation with leptin significantly enhanced MMP-2 activity resulting from MT1-MMP action, which is a known activator of the mentioned metalloproteinase. Importantly, the mRNA and protein levels of MMP-2 remained unchanged, whereas the mRNA and protein levels of MT1-MMP both increased; therefore, it may be concluded that leptin upregulates the expression of MT1-MMP, which in turn increases MMP-2 activity [166]. Moreover, leptin enhanced the synthesis of collagen type I in the myofibroblast, which contributed to increased levels of extracellular soluble procollagen type I protein in conditioned media. Total collagen synthesis was unaffected, as the level of soluble procollagen type III protein was decreased [166]. In another study by the same authors on adult rat cardiac fibroblasts, leptin was shown to regulate the actin cytoskeleton via the RhoA/ROCK pathway, and thereby induce the translocation of MT1-MMP to the membrane cell [167]. Subsequently, the creation of a cell membrane complex of CD44 with MT1-MMP enabled the cleavage of the pro-MMP-2′s propeptide domain, thus prompting MMP-2 activation [167]. The authors also determined, in a study on murine HL-1 cardiac cells, that leptin-dependent MMP-2 activation in the ECM may rather depend on p38 MAPK signaling, and not ERK1/2 nor PI3-kinase signaling [165].After leptin stimulation, signaling via p38 MAPK contributed to the reduction of TIMP-1 expression [165].

Madani et al. made a slightly different observation on human pediatric ventricular myocytes, namely, they observed the increased expression of collagen type III and IV mRNA and the decreased expression of collagen type I under leptin stimulation [164]. Interestingly, p38 MAPK and JAK/STAT signaling was involved in that process, as well as ERK1/2 [164]. However, as stated above, in murine cells, ERK1/2 signaling was not reported. Importantly, signaling via ERK1/2 was the only pathway contributing to decreased collagen type I mRNA levels [165], which may be a source of the inconsistency. In the study by Madani et al., all three signaling pathways were conducive to cardiac hypertrophy, which was evidenced by the increased expression of α-actin and myosin light chain-2 [164].

Similar conclusions were reached by da Silva-Bertani et al., who conducted their study on obese hyperinsulinemic and hypertensive rats, which were fed a high-fat diet for 34 weeks, and observed similar MMP-2 (increased) and collagen type I profiles (decreased) [127]. There was also a negative correlation between collagen type I and MMP-2 and a positive correlation between leptin and MMP-2 [127]. They also reported reduced TIMP-1 and TIMP-2 levels [127]. Low TIMP2 concentration may have enhanced the activity of MMP14 and MMP2, which in turn, cleaved collagen type I and decreased its level [127].

Leptin signaling in the myocardium is not fully understood in terms of conditions associated with leptin resistance [159]. There are numerous experimental studies on animals with a defective leptin gene (ob/ob), as well as with the impairment of its receptor (db/db or fa/fa) [27]. Similarly, as in humans, those animal models develop obesity, increased adiposity, and hyperglycemia [168]. The use of db/dbmice is also a recognized method for establishing a model of diabetes-associated cardiomyopathy, as rodents usually exhibit characteristics typical for HFpEF (such as diastolic dysfunction, cardiomyocyte hypertrophy, interstitial/perivascular fibrosis, and microvascular rarefaction) [168]. There are studies indicating that geneticallyinduced leptin-resistant animals exhibited lipid accumulation in the heart, which resulted in apoptosis [169]. This condition was reversed by the administration of leptin [169]. It was also observed that in leptin-deficient ob/ob mice, the activity of sarco(endo)plasmic reticulum (SR) Ca2+-ATPase (SERCA) and Na(+)-Ca(2+) exchanger expression were depressed [170]. This supports the fact that leptin may have an impact on the contractility of the heart muscle [170].

To conclude, it is still not known whether the impact of leptin on the myocardium in the course of obesity results from hyperleptinemia or leptin resistance; therefore, further studies are required [171]. The abovementioned studies show conflicting results regarding the influence of leptin on cardiac ECM. There is evidence documenting leptin’s matrix-degrading impact, as well as there are studies demonstrating leptin’s profibrotic properties. We may only speculate that time of exposure to obesity, different expressions of TIMPs, and differences in leptin cellular signaling may be considered cause for such inconsistency. Further research is needed to understand the complexity of leptin’s impact on the myocardium.

#### 7.3.2. Adiponectin

Adiponectin is a beneficial adipocyte-derived hormone that is involved in maintaining body energy homeostasis via suppressing gluconeogenesis in the liver and enhancing FFA oxidation in the skeletal muscles [172,173]. Such peripheral effects are due to adenosine monophosphate-activated protein kinase (AMPK) signaling and depend upon increased mitochondrial fatty-CoA import and CD36 translocation [174]. Moreover, adiponectin exhibits anti-inflammatory, antiapoptotic, and antiatherosclerotic properties [175,176] and is significantly reduced in obese individuals, leading to a state known as hypoadiponectinemia [177]. Native adiponectin may exist in three different complexes: low molecular form (LMW, trimer), middle molecular form (MMW, hexamer), and high molecular form (HMW, 12–18-mer) [175]. It is believed that HMW, which mostly exerts insulin-sensitizing properties and cardiovascular protective effects, is most profoundly decreased in obese individuals [173]. Adiponectin is believed to be an agent protective against the development of metabolic syndrome [138]. There are two major adiponectin receptors: AdipoR1 and AdipoR2 [173], whose presences have also been confirmed in the heart muscle [178]. The first is generally responsible for activating the AMPK pathway, whereas the second induces signaling through the proliferator-activated receptor alpha (PPARα) [173].

The available data suggest that adiponectin may have positive effects on the cardiovascular system and exerts antifibrotic properties. For example, in the study by Fujita et al., supplementation of adiponectin to the cultured neonatal rat cardiac fibroblasts incubated with Ang II improved the reduction of AMPK activity and partially decreased ER1/2 activity, which was formerly enhanced by AngII. Subsequently, the same authors performed a study on mice with the adiponectin gene knockout (APN-KO) and observed increased cardiac fibrosis after Ang II infusion in the APN-KO animals, when compared with the wild type (WT) controls, which was also evidenced by the increased mRNA of collagen type I, III, and TGF-β [138]. Importantly, most adverse effects were reversed after adiponectin supplementation, but not in PPARα-KO mice, which suggests the involvement of PPARα as a downstream effector of adiponectin signaling [138].

Moreover, in astudy on primary rat cardiac fibroblasts, Dadson et al. investigated that adiponectin signaling via its downstream effector APPL1 and, subsequently, AMPK activation, may exert changes in MMPs activity [179]. They observed enhanced MT1-MMP translocation to the cell membrane and increased extracellular MMP-2 activity [179].

Adiponectin deficiency may also aggravate concentric cardiac remodeling in a pressure overload condition (encountered, for example, in hypertension), which was observed in the studies on APN-KO mice [180,181]. Enhanced hypertrophy resulted from an increase in ERK and a reduction in AMPK signaling in the myocardium [180,181].

In an experiment on male SpragueDawley rats, Zhu et al. investigated whether adiponectin improved post-MI remodeling and I/R injury through AMPK-dependent and independent AMPK STAT3 activation [182]. In the model of subacute CVB3 myocarditis, APN-KO mice exhibited a lower level of MMP-9 synthesized by cardiac fibroblasts, which impaired the collagen cleavage during the myocardium injury [183].Yan et al. showed that patients affected by hypertension with a lower level of adiponectin (resulting from genetic polymorphism) were predisposed to enhanced cardiac fibrosis [184].

Although the abovementioned studies were not performed within the scope of an obesity investigation, they show how hypoadiponectinemia (which is frequently encountered in obese individuals) may have an adverse impact on the alterations in the ECM and cardiomyocyte hypertrophy and can exacerbate cardiac injury under ischemic conditions. The studies suggest that hypoadiponectinemia facilitates cardiac fibrosis; nevertheless, further research onthe settings of obesity is required to confirm the hypothesis.

**Figure 3:**
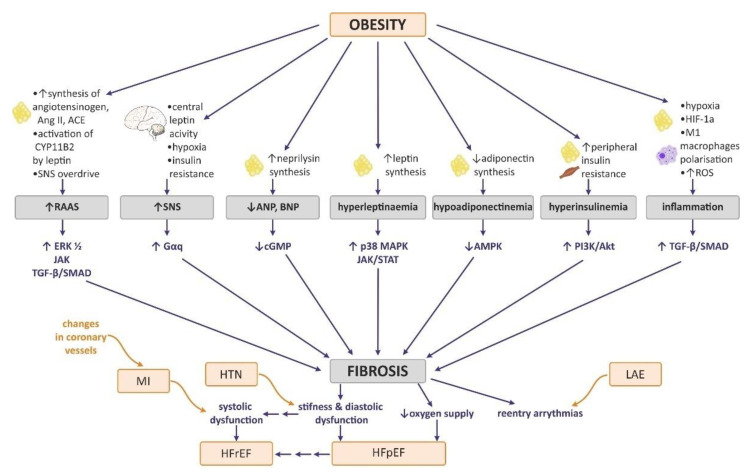
Rationale for cardiac fibrosis in the course of obesity and its clinical sequel. Ang II—angiotensin II; ACE—angiotensin-converting enzyme; SNS—sympathetic nervous system; ANP—atrial natriuretic peptide; BNP—brain natriuretic peptide; ERK1/2—extracellular-signal-regulated kinase; JAK—Janus-activatedkinase; TGF-β—transforming growth factor beta; cGMP—cyclic guanosine monophosphate; p38MAPK—p38 mitogen-activated protein kinase; JAK/STAT—Janus kinase/signal transducer and activator of transcription; AMPK—AMP-activated protein kinase; Pi3k/Akt—phosphatidylinositol 3-kinase/protein kinase B; MI—myocardial infarction; HTN—hypertension; LAE—left atrial enlargement; HFrEF—heart failure with reduced ejection fraction; HFpEF—heart failure with preserved ejection fraction.

Summary of the rationale for cardiac fibrosis in the course of obesity is presented in Figure 3.

## 8. Heart Remodeling in Selected Cardiovascular Diseases

Obesity-related metabolic alterations may trigger the development of hypertension; atherosclerosis; ischemic heart disease (IHD); and, consequently, myocardial infarct (MI). The presence of those concomitant factors may additionally change the prognosis for patients and their risk profile, as well as cardiac remodeling. On the other hand, interstitial fibrosis within the heart muscle may affect heart function, impairing the diastolic function, and therefore may be one of the causes of arrhythmia.

This section focuses on two obesity-related cardiovascular complications that have significantly different impacts on heart remodeling, namely, hypertension and myocardial infarction, as well as two major consequences of obesity-related cardiac fibrosis—heart failure and atrial fibrillation.

### 8.1. Hypertension

Hypertension (HTN), defined as elevated systolic blood pressure (SBP) ≥ 140 mmHg and diastolic blood pressure (DBP) ≥ 90 mmHg, is one of the major preventable risk factors for cardiovascular disease (CVD) and all-cause mortality worldwide [185]. Global epidemiological statistics show that the condition may pertain to about 1.28 billion adults worldwide [186]. The linkage between obesity and HTN was recognized early—specifically, at the beginning of the twentieth century [187]. It has been shown that today, up to 50–60% of obese people suffer from hypertension [44]. Even if obesity does not always induce hypertension (HTN), it may increase baseline BP and the risk of developing HTN in the future [6].

Although hemodynamic alterations (characterized by increased CO and decreased SVR) would indicate against the development of HTN, other factors such as excessive RAAs and SNS activation, hyperleptinemia, and a decrease in natriuretic peptides with consequent salt retaining, may play key pathogenic roles [8,187].

In terms of cardiac remodeling, Ang II affects the smooth muscle in the blood vessels exerting a vasoconstrictor effect, thereby increasing the afterload and pressure on the LV and prompting its concentric hypertrophy [9,135]. Enhanced RAAs activity also increases total blood volume, which further contributes to the rise in the preload [9]. Augmented aldosterone secretion from the zona glomerulosa and salt retaining may be elucidated by activation of aldosterone synthase (CYP11B2) via leptin, which was also responsible for the SNS overdrive [135].

HTN, in the absence of obesity, is associated predominantly with LV pressure overload (PO), causing concentric hypertrophy. Nevertheless, volume overload (VO) was additionally observed in obese individuals; thus, the presence of both components (PO and VO) may result in the development of either concentric or eccentric LV hypertrophy [8]. It may be also speculated that during the co-occurrence of HTN and obesity, the initial adaptive response to PO includes early development of LV hypertrophy in order to sustain even more enhanced CO. Later on, untreated HTN is followed by a slow progression to cardiac interstitial fibrosis, which impairs its diastolic function. Pertinent PO may eventually lead to dilatation, systolic dysfunction, and heart failure [70].

### 8.2. Myocardial Infarction(MI)

Acute myocardial infarction is defined pathologically as myocardial cell death due to prolonged ischemia and clinically as an ischemic-provoked acute myocardial injury accompanied by abnormalities in cardiac biomarkers [188]. MI is one of the leading causes of death globally, with a prevalence approaching three million people worldwide [189]. Obesity is a well-established modifiable risk factor for cardiovascular diseases and was also indicated in the INTERHEART study as an important contributor to the development of MI [190].

Heart remodeling after MI is complex [46]. Reparative fibrosis is the dominant type of fibrosis in post-MI remodeling and is based on replacing necrosed cardiomyocytes by a fibrotic scar [105]. Thus, infarcted areas are generally stretched and dilated, which increases the left-ventricular volume, but also exerts a combined volume and pressure load on noninfarcted tissues, leading to their hypertrophy [46]. Myocardial infarct is the predominant risk factor for HFrEF and may contribute to heart systolic dysfunction and sometimes also to heart diastolic dysfunction [191].

Cardiac remodeling after MI has been extensively studied, particularly the mechanisms on the cellular level. In the early phase, it is mostly related to inflammatory cell migration to the injured heart area. Neutrophils emerge as the first cells, as soon as 15 min after myocardial infarct, and constitute the main source of MMP-9, which cleaves the ECM scaffolding [192]. Macrophages, derived from circulating monocytes, are the subsequent source of metalloproteinases, as well as several cytokines such TNF-α, TGF-β, and IL1 [192]. They also phagocyte the necrosed material and secrete TGF-β that stimulates the fibroblast proliferation and induces collagen deposition [192]. Heart fibroblasts become activated in ischemic injury and reach their maximum proliferation within 2–4 days; later, in the first week after MI, they are differentiated into myoFBs, which secrete much more ECM than fibroblasts and provide stronger contractile support to the damaged heart [193]. Consequently, a fibrotic scar replaces the necrosed myocytes [105].

The impact of obesity on post-MI cardiac remodeling is not well-established and is quite controversial [194]. In humans, a so-called obesity paradox exists in which it is postulated that obese patients may present better long-term prognosis than lean individuals after MI despite their overall high risk for developing cardiovascular disease [195]. This hypothesis is often criticized because obese subjects with acute coronary events are generally younger than their counterparts with MI of another origin, which may represent a bias [194,196]. Importantly, the paradox may not pertain to extremely obese patients [194,196]. Interestingly, some positive impact of obesity on post-MI cardiac remodeling was also depicted in animal models with both experimentally induced obesity (usually by HFD) and MI [194,195,197,198]; therefore, they may provide some rationale for the paradox, which is discussed below.

In the study by Poncelas et al., the hybrid mouse strain B6D2F1, after 6 months of HFD, developed severe obesity and hyperinsulinemia without complications of hyperglycemia and hypertension, had improved tolerance to myocardial ischemia/reperfusion injury after MI, and long-term reperfusion in comparison with mice on a normal diet [195]. Moreover, obese mice had less LV dilatation, decreased incidence of pulmonary congestion, and improved ventricular function [195].

Heberlin et al. observed increased post-MI mortality among hyperglycemic and hyperinsulinemicKKAymice.Attenuated LV remodeling and reduced deposition of collagen type I and III occurred in the survivors; hence, reduced heart stiffness in obese survivors was detected. Changes in the ECM turnover, characterized by reduced scar formation were probably a reason for a higher rate of cardiac rupture in the early post-MI phase; however, they contributed to better overall outcome in subsequent days [199].

Inserte et al. observed a reduced infarct size in obese B6D2F1 mice submitted to transient coronary occlusion [197]. Similarly, Huisamen et al. also observed a smaller infarct size in obese Wistar rats, as well as significantly poorer early postischemic recovery than in the controls [198].

Some of the abovementioned studies focused on investigating alterations in the signaling pathways in the postischemic hearts of obese animals [194,195,198]. In those models, it was shown that hyperinsulinemia may increase PKB/Akt phosphorylation (activation) in the noninfarcted region of the hearts [194,195,197], which is believed to be protective during ischemia/reperfusion damage [200]. Nevertheless, Huisamen et al.’s study indicates the opposite as they did not observe enhancement of this signaling pathway [198].

Furthermore, Inserte et al. proposed that HFD and obesity may impact the type of substrate utilization and favor fatty acid oxidation (FAO) over glycolysis under ischemic conditions [197].

An interesting approach was undertaken by Mouton et al., as they also observed the activation of favorable signaling pathways under ischemia in obese C57BL/6J mice [194]. However, all those positive effects were abrogated in the presence of concomitant hypertension (induced by angiotensin II administration), hence the conclusion that the coexistence of untreated hypertension may more adversely impact post-MI cardiac remodeling. Obese–hypertensive animals also had reduced collagen deposition in the infarcted areas, which can cause ventricular rupture [194]. As in previous studies, obese mice had a higher mortality rate, but it was even higher in mice with concomitant hypertension [194].

There are also studies documenting the contrary observations, postulating a decreased cardiac outcome in obese rats and an increased susceptibility to post-MI cardiac dysfunction [201]. Du Toit et al. observed increased infarct size in obese rats. Interestingly, the addition of insulin to the perfusion buffer decreased the infarct size by approximately 9% in the control group and 21% in the obese animals. Conversely, the addition of FFA augmented the area at risk [201].

### 8.3. Heart Failure (HF)

Heart failure (HF) is a set of clinical symptoms (such as breathlessness, ankle swelling, fatigue) and signs (for example, elevated jugular venous pressure, pulmonary congestion), both arising from structural and/or functional abnormalities in the heart, which consequently lead to reduced CO or elevated intracardiac pressure [202,203]. The following types of HF are typically distinguished based on the value of LVEF: HFrEF (when LVEF ≤ 40%); HFpEF (when there is evidence for structural cardiac abnormalities but the LVEF is greater than 50%); and the interjacent form, heart failure with mildly reduced ejection fraction (HFmrEF), also known as heart failure with mid-range ejection fraction, (reserved for patients with a LVEF of 40% to 49%, who may benefit from the therapy administered for individuals with HFrEF) [202].

It was shown that each increment in BMI value by 1 kg/m² increases the risk of HF in females by 7% and in males by 5% (after adjustment for established risk factors) [204]. The ALLHAT study showed that HFpEF developed predominantly in individuals with a high body mass, whereas coronary heart disease contributed to HFrEF [205]. Obesity, especially when accompanied by complications such as HTN, dyslipidemia, and insulin resistance, is a risk factor for CVD, accelerates atherosclerosis progression, and increases the risk of MI, which may impair the cardiac systolic function [3,205]. However, it is HFpEF that seems to be more prevalent in obese patients, as evidenced in the recent analysis of four community-based studies [206].

The pathogenesis of HFpEF in obese patients remains less understood and is not only related to the vascular origin [156], but to several other aspects as discussed above. The role of cardiac remodeling and fibrosis may be considered as putative factors.

Increased collagen deposition in the ECM contributes to ventricular stiffness and, therefore, may impair diastolic function in obese people [207]. Cardiomyocyte hypertrophy may imply a chronic enhanced demand for ATP in the myocardium, and such maladaptation may eventually lead to heart failure [208].

The constellation of concentric hypertrophy, diastolic dysfunction, increased cardiac fibrosis, and cellular hypertrophy were presented as features characteristic ofHFpEF [150]. The current understanding of the origin of HFpEF focuses on the fact that it is the consequence of chronic inflammatory disorders, including obesity [150]. HFpEF also remains less understood and lacks a specific effective treatment [209].

### 8.4. Atrial Fibrillation (AF)

Atrial fibrillation (AF) is the most common sustained cardiac arrhythmia in humans and one of its main causes may be obesity, which is a major trigger for metabolic syndrome and diabetes mellitus [4]. Recent data suggest that AF pertains to 1% to 4% of the populations of Australia, Europe, and the USA [210]. Wanahita et al. concluded that obesity increased the risk of developing AF even by 49% [211]. The potential rationale for the increased AF rate in obese individuals includes atrial enlargement, increased fat depots, and interstitial fibrosis [1,4].

First, LAE may stimulate stretch-activated channels, paving the way for the formation of re-entry excitations. Second, infiltration of fat depots into the myocardium disrupts heart architecture leading to a slowing of the signal conduction and the maintenance of reentrant circuits. Fibrosis disrupts cell-to-cell coupling, impairs potential propagation, and also generates reentrant excitations [4,208]. Similarly, the interactions between myocytes and differentiated myoFBs may contribute to adverse electrical, mechanical, and biochemical coupling [4]. McCauley et al. also observed remodeling of the sodium, potassium, and calcium channels in their study on obese mice administered a HFD [212]. Moreover, in this study, AF was also reversed by the administration of antioxidant therapy [212].

## 9. Conclusions

Based on the available data, it is known that obesity can have an impact on heart remodeling; nevertheless, the exact mechanism of its direct effect has still not been clarified due to the multifaceted nature of different obesity phenotypes and the high incidence of other metabolic and cardiovascular diseases. On the one hand, we observe several hemodynamic changes contributing to the dilated model of heart remodeling; on the other hand, we observe a high prevalence of concentric remodeling due to concomitant hypertension, inflammation, and (neuro)hormonal disturbances—all of them putative to the development of HFpEF. The variable effect on cardiac remodeling may also be due to the type of adipose tissue distribution, the severity and duration of obesity, and the administered treatment.

Obesity significantly influences the components of the cardiac extracellular matrix and promotes interstitial collagen deposition in the heart muscle. Cardiac fibrosis may be attributable to enhanced RAAs activity, SNS overdrive, systemic inflammation and hypoxia, decreased natriuretic peptide level, hyperinsulinemia, hyperleptinemia, and hypoadiponectinemia, which are encountered in obese individuals. Unfortunately, little is known about the activity of MMPs and TIMPs in the ECM turnover and this topic should be studied more comprehensively.

The role of obesity in cardiac remodeling remains ambiguous, as the adiposity may sometimes appear to be beneficial—for example, it was shown to trigger some favorable metabolic effects under ischemic conditions.

Due to the increasing worldwide prevalence of obesity, there is an urgent need for further studies to help us understand the mechanisms of cardiac dysfunction in obese patients and to enable us to develop better diagnostic and therapeutic measurement of HFpEF.

## Figures and Tables

**Table 1:** Summary of studies performed on rodent models of high-fat diet (HFD)-induced obesity.

Investigators	Animal Model	Details of HFD Regimen	Concomitant Diseases	Indicators of Fibrosis and ECM Alterations	Cardiac Remodeling and Dysfunction
Wang et al., 2012 [125]	C57BL/6 J mice	D: 22 weeks A: 8 weeks P: 45%	glucose intolerance, hypercholesterolemia, hyperleptinemia, hypoadiponectinemia	↑Smad3, TGF-β ↓Smad1/5 and BMP-2 ↑MMP-9	↑heart weight, HW/TL, LVESD, LVEDD ↓LVEF, FS
Aurich et al., 2013 [124]	C57BL/6 J mice	D: 16 weeks A: 3 and 18 months P: 45%	hyperinsulinemia	↑fibrosis, col I, col III,	↑LV weight, BNP, cardiomyocyte hypertrophy
Guo et al., 2020 [128]	129S1/SvImJ mice	D: 16 weeks A: 8 weeks P: 60%	ND	↑fibrosis, col I, col III	↑heart weight, IVS, LVPW
Leopoldo et al., 2010 [119]	Wistar rats	D: 15 weeks A: 30 days P: 45.2%	glucose intolerance, hyperinsulinemia, hyperleptinemia, hypertension	↑fibrosis	↑LV mass, LVESD, LV wall systolic stress, PWTd
Da Silva et al., 2014 [126]	Wistar rats	D: 15 and 30 weeks A: 30 days P: 49.2% fat	glucose intolerance, hyperinsulinemia, hyperleptinemia	↑col I (15 wk), ↓col II (30 wk)	ND
Martins et al., 2015 [121]	Wistar rats	D: 20 weeks A: 30 days P: 22.7%	glucose intolerance	↑fibrosis	↑LAE, MCSAs
Eid et al., 2019 [92]	Wistar rats	D: 8 weeks A: ND P: 40% + CO	hyperinsulinemia, insulin resistance	↑TGF-β1, Smad-3, total collagen, collagen type I/III ratio, cardiomyocyte apoptosis	↑LVEDD ↓LVESD, LV contractility
Jiménez-González et al., 2020 [122]	Wistar rats	D: 7 weeks A: ND P: 35%	insulin resistance	↑fibrosis	↑heart weight, HW/TL, cardiac hypertrophy
Da Silva-Bertani et al., 2020 [127]	Wistar rats	D: 34 weeks A: 30 days P: 49.2%	glucose intolerance, hyperinsulinemia, insulin resistance, hyperleptinemia	↓col I ↑MMP-2; ↓TIMP-1, and TIMP-2	ND
Nascimento et al., 2013 [120]	Wistar Kyoto rats	D: 20 weeks A: 4 weeks P: 30%	glucose intolerance, hyperinsulinemia, insulin resistance, hypercholesterolemia, hypertriglyceridemia, hypertension	↑fibrosis	↑LVW/BW, cardiomyocyte hypertrophy
Czarzasta et al., 2018 [118]	Sprague Dawley rats	D: 12 and 16 weeks A: 4 weeks T: 31%	ND	↑fibrosis, cardiomyocyte apoptosis	ND
Hubesch et al., 2022 [129]	Sprague-Dawley rats	D: 4 and 12 months A: 4 week T:43%	glucose intolerance, hyperlipidemia, hyperleptinemia, hyperadiponectinemia	↑fibrosis	Concentric hypertrophy, ↑HW, LVSP, LVEDP, RVESP, *

HFD—high-fat diet; D—duration of the HFD regimen; A—age of rodents when onset of HFD regimen occurred; P—percentage of fat content in HFD; ECM—extracellular matrix; TGF-β—transforming growth factor β; BMP—bone morphogenic protein; MMP—metalloproteinase; HW/TL—heart weight/tibial bone length ratio; LVESD—left ventricular end-systolic dimensions; LVEDD—left ventricular end-diastolic dimension; LVEF—left ventricular ejection fraction; FS—fractional shortening; col—collagen; BNP—brain natriuretic protein; ND—no data; LV—left ventricle; PWTd—posterior wall thickness in diastole; wk—weeks; LAE—left atrial enlargement; MCSAs—myocyte cross-sectional areas; CO—corn oil; TIMP—tissue inhibitor of metalloproteinases; LVW/BW—left ventricle weight/body weight; LVSP—left ventricular systolic pressure; LVEDP—left ventricular end diastolic pressure; RVSP—right ventricular systolic pressure; *—statistically significant results only in terms of 12 months rats.

## Data Availability

Not applicable.

## Abbreviations

α-SMA, α-smooth muscle actin; ACE, angiotensin-converting enzyme; AF, atrial fibrillation; ANG II, angiotensin II; ANGPTL2, angiopoietin-like 2; AMPK, adenosine monophosphate-activated protein kinase; ANP, atrial and brain natriuretic peptide; APN-KO, adiponectin gene knockout; ATP, adenosine triphosphate; β-AR, beta-adrenergic receptor; β2-AR, β2-adrenergic receptor; BMI, body mass index; BMP, BMP—bone morphogenic protein; BP, blood pressure; BNP, brain natriuretic protein; CAD, coronary artery disease; cGMP, cyclic guanosine monophosphate; CITP, C-terminal telopeptides of collagen type I; CIIITP, C-terminal telopeptides of collagen type III; CO, cardiac output; col, collage; cTGF, connective tissue growth factor; CVD, cardiovascular disease; DAG, diacylglycerol; DBP, diastolic blood pressure; EAT, epicardial adipose tissue; ECM, extracellular matrix; EF, ejection fraction; EGF, epidermal growth factor; ERK1/2, extracellular-signal-regulated kinase; ET-1, endothelin-1; FAO, fatty acid oxidation; FAT, CD36/fatty-acid transport protein; FFA, free fatty acids; FGF, fibroblast growth factor; FS, fractional shortening; GAG, glycosaminoglycans; GSK3A, glycogen synthase kinase-3 α; GSK3B, glycogen synthase kinase-3 β; HCD, high-cholesterol diet; HFD, high-fat diet; HFpEF, heart failure with preserved ejection fraction; HFrEF, heart failure with reduced ejection fraction; HIF-1α, hypoxia-inducible factor-1 α; HMW, high molecular form; HR, heart rate; HTN, hypertension; HW/TL, heart weight/tibial bone length ratio; IHD, ischemic heart disease; IL-6, interleukin 6; JAK, Janus-activatedkinase; JAK/STAT, Janus kinase/signal transducer and activator of transcription; JNK, c-Jun N-terminal kinase; LAE, left atrium enlargement; LepR, leptin receptor; LMW, low molecular form; LOX, lisyl oxidase; LV, left ventricle; LVEDD, left ventricular end-diastolic dimension; LVEDP, left ventricular end diastolic pressure; LVEF, left ventricular ejection fraction; LVESD, left ventricular end-systolic dimensions; LVH, left ventricular hypertrophy; LVSP, left ventricular systolic pressure; LVW/BW, left ventricle weight/body weight; MAO, metabolically abnormal obese; MCP-1, monocyte chemoattractant protein-1; MCSAs, myocyte cross-sectional areas; MHO, metabolically healthy obese; MI, myocardial infarction; MMPs, metalloproteinases; MMW, middle molecular form; MT-MMPs, membrane-type metalloproteinases; myoFBs, myofibroblasts; NADPH, nicotinamide adenine dinucleotide phosphate; NEFA, nonestrified fatty acids; NITP, N-terminal telopeptides of collagen type I; NIIITP, N-terminal telopeptides of collagen type III; NO, nitric oxide; p38 MAPK, p38 mitogen-activated protein kinase; PAR-1, protease activated receptor 1; PDGF, platelet-derived growth factor; PH4, prolyl-4-hydroxylase; PI3K/Akt, phosphatidylinositol 3 kinase/protein kinase B; PICP, procollagen type I carboxy-terminal propeptide; PINP, procollagen type I amino-terminal propeptide; PKC, protein kinase C; PKG, protein kinase G; PLOD, procollagen-lysine,2-oxoglutarate 5-dioxygenase; PO, pressure overload; PPAR, peroxisome-proliferator-activated receptor; proMMPs, proenzyme form metalloproteinases; pro-TGF-β, transforming growth factor beta; PWTd, posterior wall thickness in diastole; RAAs, renin–angiotensin–aldosterone system; RF, right ventricle; Rho, Ras homologous protein; ROCK, Rho-associated coiled-coil-forming protein kinase; ROS, reactive oxygen species; R-Smad, receptor-regulated Smad proteins; RVSP, right ventricular systolic pressure; SAT, subcutaneous adipose tissue; SBP, systolic blood pressure; SERCA, sarcoendoplasmic reticulum (SR) calcium transport ATPase; SNS, sympathetic nervous system; SO, sarcopenic obese; SR, sarco(endo)plasmic reticulum; SV, stroke volume, SVR, systemic vascular resistance; TAK1, transforming growth factor beta-activated kinase 1 kinase; TIMPs, tissue inhibitor of metalloproteinases; TGF-β, transforming growth factor beta; TGFβIR, transforming growth factor-β receptor type I; TGFβIIR, transforming growth factor-β receptor type III; TNF-α, tumor necrosis factor α; TPA, tissue plasminogen activator; uPA, urokinase-type plasminogen activator; VAT, visceral adipose tissue; VCAM1, vascular cell adhesion molekule-1; VO, volume overload; WT, wild type.
